# Does sex hormone-binding globulin cause insulin resistance during pubertal growth?

**DOI:** 10.1530/EC-19-0044

**Published:** 2019-03-29

**Authors:** Shenglong Le, Leiting Xu, Moritz Schumann, Na Wu, Timo Törmäkangas, Markku Alén, Sulin Cheng, Petri Wiklund

**Affiliations:** 1 Exercise, Health and Technology Centre, Department of Physical Education, Shanghai Jiao Tong University, Shanghai, China; 2 Faculty of Sport and Health Sciences, University of Jyväskylä, Jyväskylä, Finland; 3 Medical School, Ningbo University, Ningbo, China; 4 Department of Molecular and Cellular Sports Medicine, German Sport University Cologne, Cologne, Germany; 5 The Key Laboratory of Systems Biomedicine, Ministry of Education, and Exercise Translational Medicine Center, Shanghai Center for Systems Biomedicine, Shanghai Jiao Tong University, Shanghai, China; 6 Department of Medical Rehabilitation, Oulu University Hospital, Oulu, Finland; 7 Department of Epidemiology and Biostatistics, Centre for Environment and Health, School of Public Health, Imperial College London, London, UK; 8 Center for Life Course Health Research, Faculty of Medicine, University of Oulu, Oulu, Finland

**Keywords:** sex hormone-binding globulin, insulin resistance, adiposity, puberty, menarche

## Abstract

**Background:**

The directional influences between serum sex hormone-binding globulin (SHBG), adiposity and insulin resistance during pubertal growth remain unclear. The aim of this study was to investigate bidirectional associations between SHBG and insulin resistance (HOMA-IR) and adiposity from childhood to early adulthood.

**Methods:**

Participants were 396 healthy girls measured at baseline (age 11.2 years) and at 1, 2, 4 and 7.5 years. Serum concentrations of estradiol, testosterone and SHBG were determined by ELISA, glucose and insulin by enzymatic photometry, insulin-like growth factor 1 (IGF-1) by time-resolved fluoroimmunoassays, whole-body fat mass by dual-energy X-ray absorptiometry and HOMA-IR were determined by homeostatic model assessment. The associations were examined using cross-lagged path models.

**Results:**

In a cross-lagged path model, SHBG predicted HOMA-IR before menarche β = −0.320 (95% CI: −0.552 to −0.089), *P* = 0.007, independent of adiposity and IGF-1. After menarche, no directional effect was found between SHBG and insulin resistance or adiposity.

**Conclusions:**

Our results suggest that in early puberty, decline in SHBG predicts development of insulin resistance, independent of adiposity. However, after menarche, no directional influences between SHBG, adiposity and insulin resistance were found, suggesting that observational associations between SHBG, adiposity and insulin resistance in pubertal children may be subject to confounding. Further research is needed to understand the underlying mechanisms of the associations between SHBG and cardiometabolic risk markers in peripubertal children.

## Introduction

Sex steroids are important regulators of pubertal development and their biological action is governed by sex hormone-binding globulin (SHBG) (1). Serum SHBG levels rise from birth to early childhood, then decline in early puberty, and thereafter start to rise again until early adulthood (2, 3, 4). The mechanism for the decline in SHBG during puberty is not clear, but is likely driven by other factors in addition to sex steroids since SHBG levels also decline in boys with idiopathic hypoandrogenism and in precocious pubertal girls treated with gonadotropin-releasing hormone analogs (4, 5). Some of the variation in circulating SHBG might be related to body composition and insulin sensitivity, both of which change substantially during puberty (6, 7). Along these lines, low serum SHBG level has been associated with increased adiposity and insulin resistance in children and adolescents (3, 8, 9, 10, 11, 12); therefore, it has been hypothesized that SHBG might be an important regulator of puberty and a biomarker for cardiometabolic risk (3). However, confounding or reverse causation, may explain part of the association. Indeed, there may be a bidirectional relationship between SHBG, insulin resistance and adiposity. However, no study has examined the reciprocal relationship of these variables from childhood across puberty, and thus, the causal relations between these variables remain unclear.

In the present study, we investigated bidirectional associations between serum SHBG, adiposity and insulin resistance at five time points in females transitioning from pre-puberty to early adulthood using a cross-lagged panel model analysis. In this way, we aimed to elucidate whether increased adiposity and insulin resistance during pubertal transition predicts subsequent serum SHBG or vice versa.

## Subjects and methods

### Study design and participants

The study subjects originated from a longitudinal study which has been described previously (13, 14). Briefly, 396 girls were recruited from local schools in the city of Jyväskylä and its surroundings in Central Finland to participate in a longitudinal study of determinants of body composition during pubertal growth (the Calex study). Girls in Tanner stage 1 and 2 were included at the baseline (13, 14). Data collection was performed at baseline and after 1, 2, 4 and 7.5 years. In order to avoid seasonal effects, all information was collected and laboratory tests (included the blood sample collection) were performed within a 2-week period during the same month (January to February) at each assessment wave. For the purpose of the present report, we excluded 13 girls who reportedly used oral contraceptives at the age of 18 years, due to the influence of oral contraceptives on sex hormone and SHBG concentrations (1). Thus, the total number of subjects was 258 girls at baseline, 202 girls at the 1-year follow-up, 222 girls at the 2-year follow-up, 118 girls at the 4-year follow-up and 236 girls at the 7.5-year follow-up assessments, respectively.

The age at menarche was defined as the first onset of menstrual bleeding reported by questionnaire or phone call during the follow-up. Time relative to menarche (TRM, in months) was defined as the difference between the age at menarche and the measurement time points. Thus, 100, 81, 38, 11 and 0% of the girls were premenarcheal at baseline and 1, 2, 4 and 7.5 years, respectively. Current health status and regular medications were checked by study nurse and physician. Lifestyle and behavioral characteristics as well as medical history were collected using validated self-administered questionnaire. Girls under 15 years of age filled in the questionnaire with their guardians’ assistance, after which all the questionnaires were checked by a study nurse. The study protocol was approved by the Ethical Committees of the University of Jyväskylä, the Central Hospital of Central Finland and the Finnish National Agency of Medicines (memo 22/8/2008). The participants and their legal guardians provided written consent prior to the laboratory tests.

### Anthropometric and body composition assessment

All measurements were performed after an overnight fast. Participants’ weight was determined with an accuracy of 0.1 kg for each subject using an electronic scale which was calibrated before each measurement session. Dual-energy X-ray absorptiometry (Prodigy, GE Lunar Corp., Madison, WI, USA; software version 9.3) was used to measure whole body fat mass (FM) at baseline, 2- and 7.5-year follow-up. Precision of the repeated measurements of dual-energy X-ray absorptiometry expressed as coefficient of variation was 2.2% for FM.

### Biochemical measurements

Blood samples were collected in the morning between 07:00 and 09:00 h after an overnight fast at each time point. In girls with regular menses, blood sampling was performed in early follicular phase (between 2 and 5 days after the initiation of the menstrual bleeding) (13). Serum was extracted by centrifugation and stored immediately at −80°C until analysis. The samples from different time points were analyzed by one technician using the same kits and instrument. Estradiol (E2), testosterone and SHBG were determined by ELISA (NovaTec Immunodiagnostica GmbH, Dietzenbach, Germany). Inter- and intra-assay coefficients of variation were 3.2 and 5.4% for E2, 3.9 and 6.2% for testosterone and 1.1 and 1.1% for SHBG, respectively. Fasting plasma glucose was analyzed using the KONELAB 20XTi analyzer (Thermo Fischer Scientific Inc.) and fasting serum insulin was determined by immunofluorescence using the IMMULITE Analyzer (Diagnostic Products Corporation, Los Angeles, USA). Insulin-like growth factor 1 (IGF-1) was assessed using time-resolved fluoroimmunoassays (IMMULITE, Siemens Healthcare Diagnostics). The homeostatic model assessment of insulin resistance index (HOMA-IR) was calculated as fasting insulin concentration*fasting glucose concentration/22.5 (15).

### Statistical analysis

Shapiro–Wilk test was used to check normality of the data using IBM SPSS Statistics for Windows, version 22.0 (IBM Corp). If data were not normally distributed, their natural logarithms were used. Pearson correlation coefficients were used to assess the relationship between SHBG and adiposity, HOMA-IR controlling for T and E2 and between SHBG and HOMA-IR controlling for BMI for each time point separately. The Fisher’s Z transformation test was performed (taking into account the differences of number of subjects at different time points) to make the correlations between SHBG and HOMA-IR at different time points comparable.

A hierarchical nonlinear model with random effects was used to assess the temporal patterns of SHBG from pre-puberty to early adulthood (MLwin 2.20 software, Multiple Project, Institute of Education, University of London, UK). Age was entered as the explanatory variable in the form of polynomial spline functions to explain the change of target variables over time.

To assess the direction of effect between SHBG and insulin resistance from childhood to early adulthood, a bivariate cross-lagged panel model was used to estimate the structural relations of repeatedly measured variables at five different time points. The auto-regressive part of the model indicates the temporal stability of the variables from one time point to the next, while cross-lagged paths are used to assess reciprocal relationships between the variables at consecutive time points, that is, SHBG and insulin resistance (HOMA-IR) during the follow-up. The full cross-lagged path model is shown in Supplementary Fig. 1 (see section on supplementary data given at the end of this article). Model fitting was conducted with Mplus version 7 (16).

## Results

**Figure 1:**
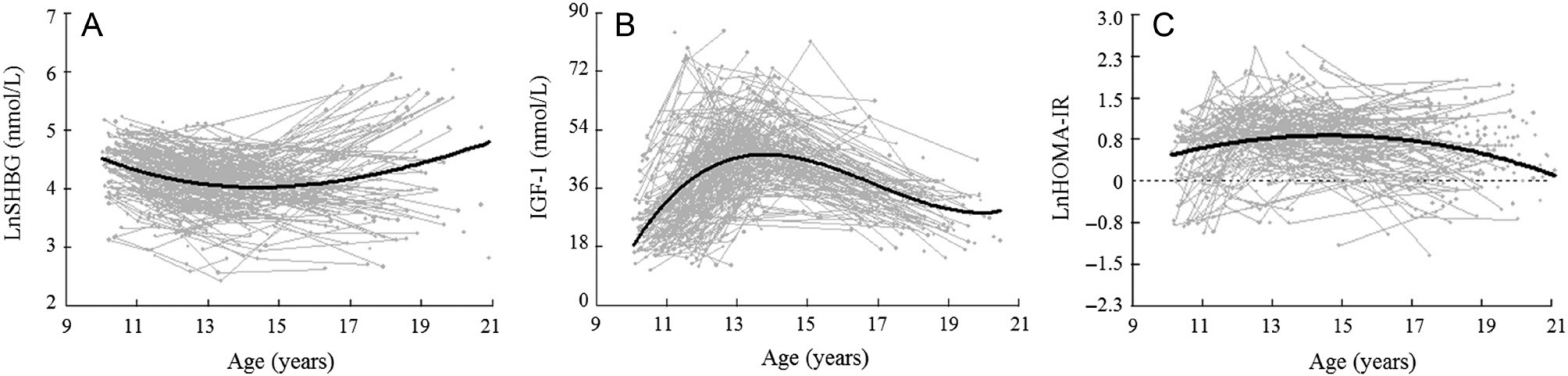
Temporal pattern for SHBG (A), IGF-1 (B) and HOMA-IR (C) levels from premenarche to early adulthood. Data for SHBG are plotted against age. Gray lines with dots represent longitudinal change of each individual and the black line is the best fitting line derived from the hierarchical models. The values on the y-axis are back-transformed from natural log SHBG and HOMA-IR.

General characteristics of the study population are presented in Table 1. More detailed information on anthropometrics and adiposity, testosterone and E2 have been presented in our previous studies (13, 14, 17). Briefly, FM increased significantly from childhood to early adulthood. Before menarche, levels of E2 and T increased, and E2 reached a plateau slightly prior to menarche (13, 14). After menarche, E2 remained at a relatively high level, while testosterone kept increasing at a lower rate up to 18 years of age (13, 14). Change of the concentrations of SHBG decreased until menarche (13-year old), maintained at a lower level until approximately 1 year after menarche and increased steadily thereafter into early adulthood (Fig. 1A). On the other hand, IGF-1 rapidly increased until menarche and decreased thereafter (Fig. 1B), while HOMA-IR increased before and around menarche and decreased thereafter returning to baseline level in early adulthood (Fig. 1C).

**Table 1:** General characteristics at different measurement time points in peripubertal girls.

	Baseline (*n* = 258)	1-year follow-up (*n* = 202)	2-year follow-up (*n* = 222)	4-year follow-up (*n* = 118)	7.5-year follow-up (*n* = 236)
Age, years	11.2 (0.8)	12.1 (0.7)	13.2 (0.7)	14.7 (1.0)	18.3 (1.1)
Height, cm	146 (8)	152 (8)	158 (7)	163 (6)	166 (6)
Weight, kg	39.1 (8.7)	44.1 (10.3)	50.0 (10.5)	55.9 (11.4)	60.3 (10.0)
BMI, kg/m^2^	18.3 (2.9)	19.1 (3.4)	20.0 (3.5)	21.0 (3.8)	21.9 (3.1)
FM, kg	10.7 (5.6)	–	13.8 (7.3)	16.0 (8.5)	19.3 (7.4)
SHBG, nmol/L	82.3 (32.6)	69.5 (29.4)	64.4 (28.2)	63.4 (29.5)	125 (119)
Testosterone, nmol/L	0.50 (0.5)	0.69 (0.5)	1.26 (0.7)	1.93 (2.0)	5.02 (7.8)
E2, nmol/L	0.11 (0.1)	0.16 (0.1)	0.17 (0.1)	0.26 (0.3)	0.46 (0.9)
Glucose, mmol/L	5.5 (0.4)	5.2 (0.5)	5.4 (0.5)	5.3 (0.6)	5.3 (1.2)
Insulin, μU/mL	9.0 (9.5)	12.1 (7.1)	12.7 (9.1)	10.4 (6.7)	8.3 (4.6)
HOMA-IR	1.67 (0.8)	2.66 (1.3)	2.88 (1.8)	2.41 (1.5)	1.94 (1.1)

Data are given as mean and their standard deviation. Whole body fat mass was not assessed at the 1-year follow-up by DXA.

BMI, body mass index (weight (kg)/height (m)^2^); E2, estradiol; FM, whole body fat mass; HOMA-IR, the homeostatic model assessment of insulin resistance index; SHBG, sex hormone-binding globulin.

Cross-sectional associations between SHBG and BMI, FM and HOMA-IR are shown in Table 2. After controlling for E2 and T, SHBG was inversely correlated with BMI (*P* < 0.01 for all) at each time point and FM at baseline (*P* < 0.01), 2-year follow-up (*P* < 0.01), 4-year follow-up (*P* < 0.01) and 7.5-year follow-up (*P* < 0.05). Negative associations of SHBG with HOMA-IR were found at baseline (*P* < 0.05), 1-year follow-up (*P* < 0.01), 2-year follow-up (*P* < 0.01) and 4-year follow-up (*P* < 0.05), but not at 7.5-year follow-up. In addition, controlling for BMI, the negative association between SHBG and HOMA-IR remained significant only at 1-year follow-up (*P* = 0.005). However, after controlling for serum triglycerides, the associations remained significant for 1-year follow-up (*P* < 0.001), 2-year follow-up (*P* = 0.012) and 4-year follow-up (*P* = 0.005) but disappeared for the baseline and the 7.5-year follow-up.

**Table 2:** Cross-sectional associations between SHBG and BMI, FM and HOMA-IR at different follow-up time points (*r* values were standardized by Fisher’s *Z* transform test).

	SHBG				
	Baseline	1-year follow-up	2-year follow-up	4-year follow-up	7.5-year follow-up
BMI	−0.659**	−0.751**	−0.712**	−0.642**	−0.313**
FM	−0.714**	–	−0.678**	−0.575**	−0.237*
HOMA-IR	−0.190*	−0.488**	−0.304**	−0.313*	−0.006

Data shown are partial correlation coefficients for obesity, insulin resistance with SHBG controlling for testosterone and E2. Natural logarithm transformed data were used for the analysis.

*P < 0.05, **P < 0.01.

BMI, body mass index (weight (kg)/height (m)^2^); FM, whole body fat mass; HOMA-IR, the homeostatic model assessment of insulin resistance index; SHBG, sex hormone-binding globulin.

**Figure 2:**
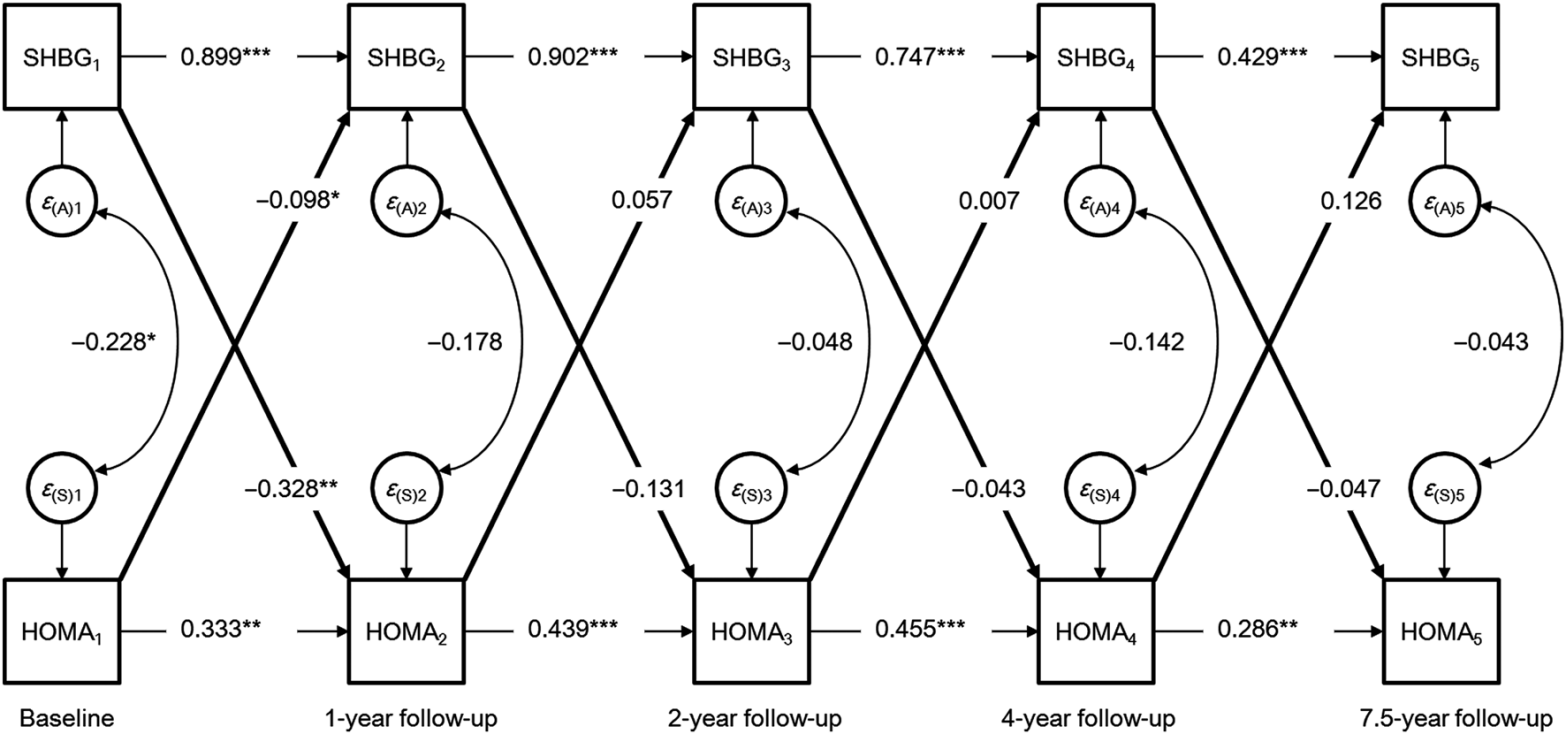
Cross-lagged path model for SHBG and HOMA-IR. *P < 0.05, **P < 0.01, ***P < 0.001.

The cross-lagged panel model showed that SHBG predicted subsequent SHBG at each time point (*P* < 0.001 for all, Fig. 2). Similar patterns were observed between the repeated measurements of HOMA-IR, albeit with lower effect sizes (*P* < 0.05 for all). This auto-regressive part of the model indicates that the temporal stability of SHBG from pre-puberty to early adulthood is higher than the temporal stability of HOMA-IR over the same time period. The cross-lagged model also showed that baseline SHBG predicted HOMA-IR (β = −0.328 (95% CI: −0.520 to −0.136), *P* = 0.001) at 1-year follow-up, vice versa baseline HOMA-IR predicted SHBG at 1-year follow-up (β = −0.098 (95% CI: −0.191 to −0.004), *P* = 0.040). We also added IGF-1 into the cross-lagged model to test the effects of GH/IGF1 axis on the associations, but the results remained virtually unchanged.

To assess whether menarche status moderated the relationship between SHBG and HOMA-IR, we performed a sensitivity analysis by omitting all girls who were post-menarcheal at 1-year follow-up (19% of the total sample). When only pre-menarcheal girls were included in the analysis, baseline SHBG still predicted HOMA-IR (β = −0.320 (95% CI: −0.552 to −0.089), *P* = 0.007) but baseline HOMA-IR did not predict SHBG at 1-year follow-up (β = −0.045 (95% CI: −0.137 to 0.047), *P* = 0.336).

To assess whether the associations were mediated by increased adiposity, we adjusted for BMI and found that baseline SHBG still predicted HOMA-IR (β = −0.296 (95% CI: −0.564 to −0.028), *P* = 0.031), but HOMA-IR did not predict SHBG at 1-year follow-up (β = −0.043 (95% CI: −0.108 to 0.040), *P* = 0.367). No directional influence between SHBG and HOMA-IR was found in subsequent time points after menarche. The model had an excellent fit to the data (χ^2^ = 45.39, *P* = 0.005, confirmatory fit index = 0.973, root mean square error of approximation = 0.048, for SHBG and HOMA-IR).

Similarly, no evidence for significant directional influence was found between SHBG and BMI during the follow-up (Supplementary Fig. 2). Because BMI is not the optimal measure of adiposity in children and adolescents, we also assessed the cross-lagged associations between SHBG and whole-body FM at baseline, 2-year follow-up and 7.5-year follow-up (Supplementary Figs 3 and 4). Similar to BMI, no directional influence was found (Supplementary Fig. 4). The model had an excellent fit to the data (χ^2^ = 49.02, *P* = 0.002, confirmatory fit index = 0.988, root mean square error of approximation = 0.052, for SHBG and BMI).

## Discussion

This longitudinal study across pubertal years showed that serum SHBG was inversely associated with adiposity and HOMA-IR, and this pattern was independent of sex steroids. However, no evidence was found that the variance and temporal trends in SHBG would be explained to a significant degree by increased BMI. Conversely, directional influence was observed between SHBG decline and HOMA-IR development before menarche, independent of BMI, whereas after menarche no evidence for directional influence was found in either direction.

Puberty is a period of dynamic physiologic, hormonal and metabolic changes, including activation of the reproductive axis and subsequent secretion of sex steroids, accumulation of both fat and fat-free mass and transient decrease in insulin sensitivity (18). Our study confirm these well-known pubertal-related patterns and further demonstrates temporal stability in SHBG and insulin resistance levels from childhood to early adulthood, a phenomenon that is likely explained partly by genetic factors (19, 20, 21), and/or early life programming (22, 23). The level of sex hormones and SHBG in our study are largely in accordance with earlier studies (3, 4, 10, 24); however, compared with other studies, we found slightly lower absolute concentrations of estradiol in post-menarcheal girls. Such discrepant findings may be attributable to study design as blood samples in our study were collected in a strictly defined period in early follicular phase in all girls with regular menses, whereas in previous studies blood sampling were not timed to a specific phase of the menstrual cycle. Inter-individual variation and differences in methodology such as sample preparation and difference in affinities of the antibodies used in different assays can also account for the small differences in sex hormones concentrations.

Growing evidence indicates that low circulating SHBG level is an indicator of adiposity and insulin resistance and therefore SHBG may be a clinically useful biomarker for the early identification of children who at risk to develop obesity-related metabolic disorders (25). This evidence comes mainly from cross-sectional investigations in peripubertal children (4, 26), and studies that have found weight loss to be associated with decreased insulin resistance and increase in serum SHBG level (27). The underlying mechanism for this association remains unclear but one explanation may be that increased lipogenesis regulates SHBG gene expression through altering hepatocyte nuclear factor 4 alpha (HNF-4α) levels (28). In line with this, several HNF4α genetic variants have been associated with obesity-related metabolic disorders in children and adolescents (29). However, whether the HNF4α alleles link low SHBG levels to obesity and metabolic disorders remains unclear.

Longitudinal studies that have assessed temporal associations between SHBG, adiposity and insulin resistance from childhood to adulthood are few and far between. Baer *et al.* examined prospective associations between adiposity and circulating levels of sex hormones among 286 girls followed from childhood (8–10 years at baseline) to early adulthood and found that circulating SHBG was inversely associated with BMI (24). Similarly, Pinkney *et al.* reported negative associations between SHBG, BMI, and fasting insulin in a longitudinal study of 347 children from age 5 to 15 years (3), and Glueck *et al.* showed that low SHBG levels in girls at the age of 14 predicted development of metabolic syndrome 10 years later (30). Our results complement the above-cited studies by demonstrating inverse associations between serum SHBG, adiposity and insulin resistance from childhood to early adulthood. However, the bivariate cross-lagged panel analysis indicated that earlier SHBG level did not predict the level of adiposity at subsequent time points after menarche, or the other way around. Interestingly, we found that SHBG predicted HOMA-IR before menarche, while after menarche no directional influence in either direction was found. Controlling for BMI, the significance remained. These findings suggest potential causal predominance of decline in SHBG level on HOMA-IR development in early puberty, but that only a very small portion of the variance and temporal trends in serum SHBG during pubertal growth is explained by increased insulin resistance or vice versa. Thus, it may be that the associations found in previous observational studies in peripubertal children may have been subject to unmeasured confounding (3, 4, 24, 26, 30).

We acknowledge that our study cannot prove or disprove causal relationships; but because appropriate temporal order is a pre-requisite for causality, our longitudinal study with several follow-up waves can provide information about the directional influence of variables have on each other over time. The reason why directional influence was observed only before menarche and not after is not clear. It could be explained by the myriad of hormonal and metabolic changes and transient decrease in insulin sensitivity that occur during puberty. However, since the influence of one variable on another may be a function of time between waves of measurement, we cannot rule out the possibility that the lack of directional influence was attributed to longer length of time between 2-year, 4-year and 7.5-year measurement waves. If the time between measurements is too long, the effects can dissipate before the next time of measurement. However, confirming these findings are difficult because direction of effects between SHBG and cardiometabolic risk factors have not been examined in earlier longitudinal studies in children and adolescents. On the other hand, a recent large, population-based study in young adults found that SHBG was associated with multiple circulating metabolites reflecting the degree of adiposity and insulin resistance, but the Mendelian randomization analyses suggested weak causal effects (31), which support the idea that the results found in earlier observational studies may have been partly confounded. Thus, the potential involvement of SHBG in the etiology of metabolic deviations related to cardiometabolic diseases remains unclear. Further research is needed to understand how SHBG is regulated in children and whether sex hormone binding to SHBG or other endocrine factors may underpin the observed associations of SHBG with obesity, insulin resistance and other cardiometabolic risk markers in peripubertal children.

Our study has both strengths and limitations. The longitudinal analysis was conducted in a homogenous nationally representative cohort of healthy females followed from childhood across puberty into early adulthood with several follow-up waves. Rigor was also exhibited in collecting blood samples in a strictly defined period of the menstrual cycle in girls with regular menses, which minimizes the variance in intra and inter-individual sex steroid concentrations. Bivariate cross-lagged path analysis was used to explore the direction of the associations between SHBG, adiposity and insulin resistance, which allows examining temporal associations better than simple regression analysis. In the present study, hormone measurements during the follicular phase may have underestimated the production of estradiol across the menstrual cycle because estradiol levels during the follicular phase are at the lowest level of the cycle. Therefore, the longitudinal changes of SHBG being independent of sex steroids must be viewed as tentative. However, as mentioned in the methods, blood samples collected within a 2-week period during the same month at each assessment wave, thus limiting seasonal, intra and inter-individual variation in hormone levels. In addition, levels of estradiol and testosterone were assessed by immunoassay, and low levels characteristic of normal girls are known to be overestimated when determined with immunoassay (e .g. the level of estradiol (0.11 nmol/L)) in Table 1 compared to the values in prepubertal girls by LC-MS (32). Finally, the study participants were all females and therefore caution should be taken in seeking to generalize from our results to boys.

In summary, our study suggests potential causal association between decline in SHBG level and development of insulin resistance before menarche, but not after. Further research is needed to understand the underlying mechanisms of the associations between SHBG and cardiometabolic risk markers in peripubertal children.

## Supplementary Material

Supplementary Figure 1. Conceptual model for assessing cross-lagged associations between serum SHBG and adiposity and HOMA-IR. For i < j, Xi -> Xj and Yi -> Yj are the autoregressive coefficients (tracking over time) and Xi -> Yj andYi -> Xj are the cross-lagged coefficients. d = residual variance.

Supplementary Figure 2. Cross-lagged path model for SHBG and BMI. * p < 0.05, ** p < 0.01, *** p < 0.001.

Supplementary Figure 3. Conceptual model for assessing cross-lagged associations between serum SHBG and FM. For i < j, Xi -> Xj and Yi -> Yj are the autoregressive coefficients (tracking over time) and Xi -> Yj andYi -> Xj are the cross-lagged coefficients. d = residual variance.

Supplementary Figure 4. Cross-lagged path model for SHBG and FM. *** p < 0.001.

## Declaration of interest

The authors declare that there is no conflict of interest that could be perceived as prejudicing the impartiality of the research reported.

## Funding

This work was supported by the Academy of Finland (grant no. 24302031 and 135038), Ministry of Education of Finland, University of Jyväskylä, Natural Science Foundation of Zhejiang Province (No. LY16H070001), China State Sport General Administration (2015B039), the Nature Science Foundation of China (31571219) and EVO research grants 2012/2013 from Oulu University Hospital. Timo Törmäkangas was funded by the Academy of Finland (No. 286536). Shenglong Le was funded by the International Mobility CIMO (No. KM-17-10469).
