# Supplementary figures and images for: Does sex hormone-binding globulin cause insulin resistance during pubertal growth?
Source: Endocr Connect. 2019 Mar 29;8(5):510–7. doi: 10.1530/EC-19-0044 (PMC6499923; doi:10.1530/EC-19-0044)

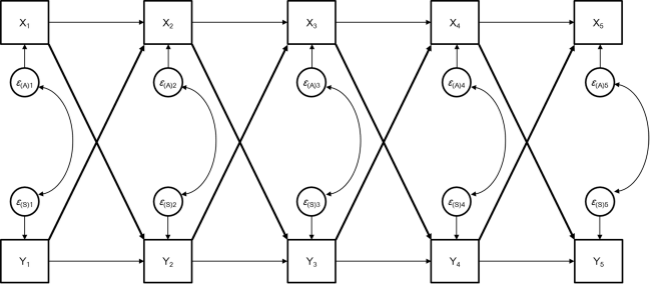

Baseline

1-year follow-up

2-year follow-up

4-year follow-up

7.5-year follow-up

Supplement: Supplementary Figure 1. Conceptual model for assessing cross-lagged associations between serum SHBG and adiposity and HOMA-IR. For i < j, Xi -> Xj and Yi -> Yj are the autoregressive coefficients (tracking over time) and Xi -> Yj andYi -> Xj are the cross-lagged coefficients. d = residual variance. [file supplementary_figure_1.pdf]

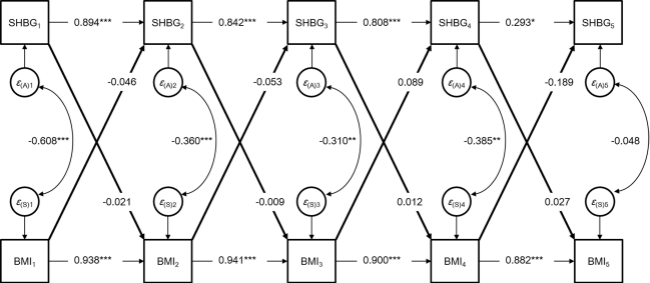

Baseline

1-year follow-up

2-year follow-up

4-year follow-up

7.5-year follow-up

Supplement: Supplementary Figure 2. Cross-lagged path model for SHBG and BMI. * p < 0.05, ** p < 0.01, *** p < 0.001. [file supplementary_figure_2.pdf]

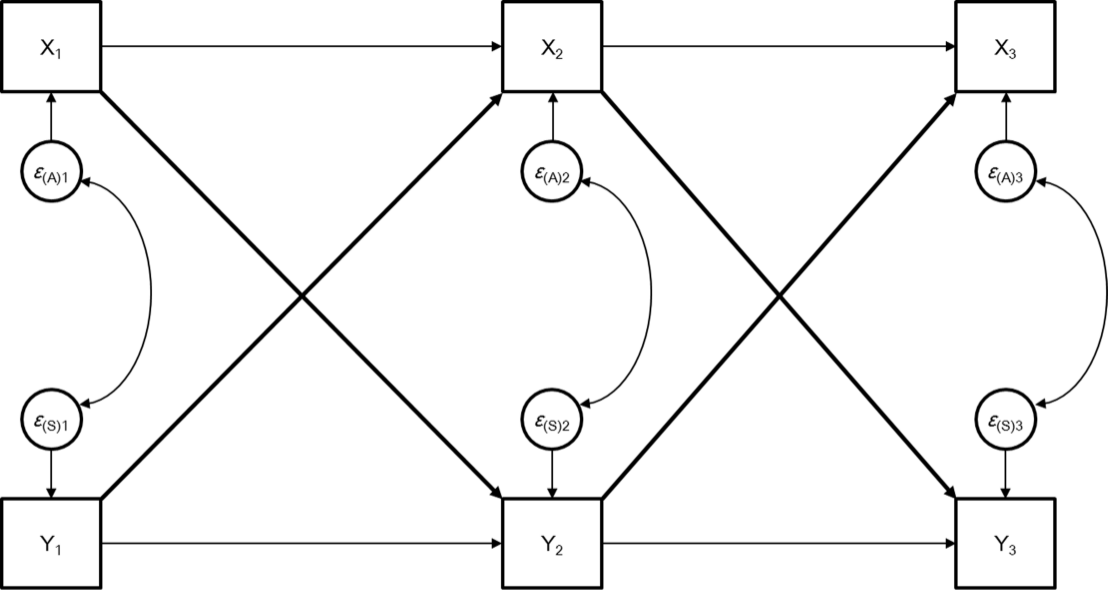

Baseline

2-year follow-up

7.5-year follow-up

Supplement: Supplementary Figure 3. Conceptual model for assessing cross-lagged associations between serum SHBG and FM. For i < j, Xi -> Xj and Yi -> Yj are the autoregressive coefficients (tracking over time) and Xi -> Yj andYi -> Xj are the cross-lagged coefficients. d = residual variance. [file supplementary_figure_3.pdf]

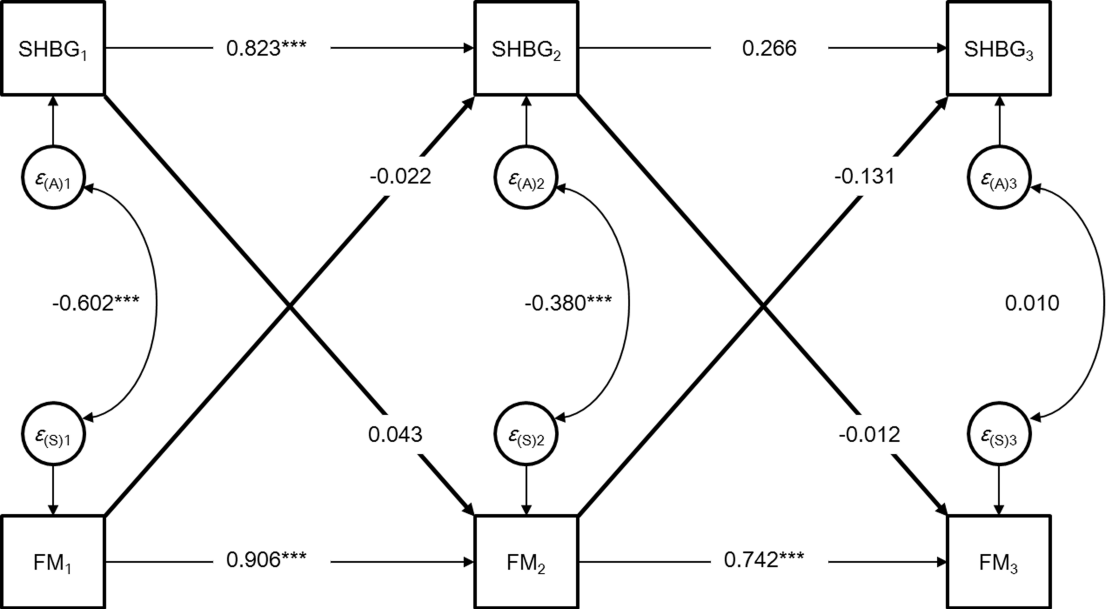

Supplement: Supplementary Figure 4. Cross-lagged path model for SHBG and FM. *** p < 0.001. [file supplementary_figure_4.pdf]
